# Medical Jurisprudence

**Published:** 1825-10-01

**Authors:** 


### MEDICAL JURISPRUDENCE.

1. *Contagion.** Dr. Maclean will now, we hope, feel compensated for any parliamentary defeat or professional neglect which he may have experienced in this country, by the attention which his works have received from some most "learned Thebans" abroad. Thousands of indexes have been ransacked in Germany and Italy to prove, what no man in his senses doubts the existence of contagion in plague and in fevers. The fact of the existence or non-existence of contagion, would appear to most rational beings the main point to be ascertained. But this is not the case. The great question now is—whether or not the existence of contagion was thought of or believed two thousand years ago? To prove or disprove this point, numerous volumes have been written, and the pages of medical journals, magazines, and even newspapers filled to satiety. We confess (to our shame no doubt) that we are somewhat costive of our pages and our time in such investigations—both happening to be otherwise more profitably employed. Our notice, therefore, of the litigation under consideration will be brief, and in proportion to the estimation which we form of its intrinsic importance.

We averred, in our last Number, that Dr. Maclean had made very few converts among his professional brethren in this country. He, however, has some disciples—and so would Mahomet or Beelzebub, were they to appear once more on the scene. But, although few—very few, paid any attention to Dr. Maclean's extravagant denial of the contagion of plague, yet his confident assertions and apparently laborious researches respecting the comparatively recent origin of the doctrines of contagion, staggered many people who had not leisure or inclination to wade through an ocean of ancient volumes, in search of information on this particular point. Some of our own countrymen, however, especially the learned Dr. Yeats, did take the trouble of pointing out the erroneous statements of the arch-anticontagionist; but this had not the slightest effect on *him*. His policy and that of his followers is to reiterate incessantly the same statements and opinions, no matter whether true or groundless, so as to tire out opposition, and induce belief, not by strength of argument but by dint of repetition.

Our German and Italian brethren (who often form most ludicrous ideas of what is going forward in this country) conceiving that Dr. Maclean had nearly inoculated all England with his peculiar doctrines, have seriously set about controverting his opinions, and subverting what he calls his *facts*, in certain tomes of recondite lore, of which our respected cotemporary of the North has given a copious account last quarter. For ourselves, we shall be much more brief—partly because we attach much less importance to the subject than some of our neigh-

* Dr. Marx—Dr. Omodei—Edinburgh Medical and Surgical Reviewer—*versus* Dr. Maclean and *his Disciples*.

bours—and partly because the labours of our cotemporaries have now rendered diffuseness on this point unnecessary.

It is well known that Dr. Maclean asserts, that no trace of the doctrine of contagion is to be found in the ancient authors, and that the said doctrine was first promulgated as a state trick, or Popish plot, about the middle of the sixteenth century. It has been well observed by our Northern cotemporary, that the very circumstance of removing the Council of Trent to Bologna, *on the plea of contagion,* proves, in the mind of every unbiassed man, that a belief in the existence of contagion had previously been general or popular. Why attempt to frighten people by a thing of which they could have no knowledge, or with the danger of which they could not at all be acquainted?—Suppose a fever were now to arise in London, and Ministers wished to frighten away the Parliament by representing the said fever as possessing a strange property never before heard of or conjectured—such, for instance, as the power of converting a man's gold into brass—or of changing his three per cent. Consols into Greek Scrip.:—how would the Humes and Broughams and Burdetts laugh at such a bug-bear?—yet such a bug-bear must have been the doctrine of contagion, if no idea of its existence occurred previously to the translation of the Council of Trent.

But we shall have better data than mere reasoning to go on presently.

We shall pass over the customs of the Egyptians—the diseases on the banks of the Serbonis, as described by Plutarch—the flight of the Israelites, supposed to be polluted with itch and leprosy—and the consecration of the Ibis, as quite futile, if not ridiculous arguments respecting the belief of contagion in Egypt. That the JEWS, however, believed in contagion, we have "proofs of Holy Writ"—witness the minute directions in Leviticus for purifying lepers, and cleansing houses supposed to be infected with the plague of leprosy or scall. Nothing can be more clear than that these ceremonies were based on a popular belief of the communicability, from person to person, of these disorders.

Among the Greek authors the traces of belief in contagion are very unsatisfactory. Hippocrates no where alludes to it. But although the doctrine is not to be found in the medical writings of the Greeks, Romans, or Arabians, yet it is very certain that the historians and philosophers of those times have recorded their belief in it—and that unequivocally. Omodei quotes the authority of Thucydides, Aristotle, Dionysius of Halicarnassus, Livy, and several others, in support of this position.—The description of the plague of Athens by Thucydides is well known; but it is not supposed to be so much to the point as the event which happened at the siege of Potidœa, a town in Macedonia, where it is represented by the abovementioned historian, that the troops of Theopompus became affected with a fatal disease caught from those under another leader, Agnon. There are passages in Plutarch respecting the plague of Athens, which are supposed to imply a belief in its contagious nature; but they are equivocal. One hundred years afterwards, Aristotle proposes as a matter of enquiry, why pestilence, of all diseases, affects, at one time, only those who approach the sick—and at another time, a whole community indiscriminately? Dionysius of Halicarnassus records three visitations of pestilence among the Roman people; but we confess that several of the passages quoted by Marx are very far from satisfactory. One, however, distinctly alludes to the conveyance of a disease by means of animals. Thus, he says, the peasantry were affected by the sheep and other animals with which they lived in daily intercourse.

Appian, the Greek historian, who flourished more than a century before the Christian Era, describes an epidemic arising from noxious exhalations in Illyria. The inhabitants of the stricken district fled from their habitations carrying the disease with them, and were refused admittance in healthy situations, for fear of their communicating the pestilence. From this time till nearly 200 years after the Christian Era, there is a hiatus in regard to contagion. Dio Cassius then relates the visitation of a pestilence at Rome, which was of singular fatality. But what is most to the point is the record of a diabolical practice then pursued —namely the destruction of people in various and even distant parts of the Roman Empire, by means of pricks from needles dipped in the pestilential poison.

Eusebius, bishop of Cæsarea, A.D. 340, describes a plague in Alexandria, where the Christians, through humanity, performed kindly offices among the sick, and were often, in consequence, smitten with the epidemic; whereas the Gentiles, from terror of the contagion, abandoned the sick, or even thrust them out of doors!*

Father Gregory of Nyssa, was, in his day, the archetype of Dr. Maclean himself—though of a very different stamp. He was a vehement anti-contagionist, and exhorted his flock not to believe in the communicability of pestilential diseases. His doctrines had a humane and benevolent object in view. They did honour to his heart, at least, though probably not to his head. At all events they prove the belief in contagion at that time, just as unequivocally as the volumes of Dr. Maclean would prove the present belief, should they be turned up some fifteen hundred years hence by another contagionist or anti-contagionist.

Five centuries and a half after the birth of Christ, a terrific pestilence scourged almost the whole of the then known surface of the habitable globe. Nobody, now a days, would imagine that such an epidemic could *arise* from contagion;—but few sensible or observant physicians could entertain any doubt that innumerable sources of contagion must have been formed during the prevalence of such universal sickness.

* We may here remark, once for all, that we do not bring forward Dr. Marx's researches as proofs of actual contagion, but of the belief in its existence among the Ancients—a fact denied in the strongest language by Maclean and his disciples. The Gentiles may have been wrong in their opinions, and certainly they were most culpable in their conduct; but the record of their inhumanity is a proof of their credence in the doctrine of contagion.

Hence we cannot wonder that two historians, Evagrius and Procopius, should have maintained diametrically opposite opinions—one, that the disease was contagious—the other, that it was destitute of any such character. They had, both of them, materials and grounds for their opinions. Both of them were ultras, or exclusionists—and both of them were wrong, as is the case to the present hour. The testimonies on both sides, however, unequivocally prove that belief in contagion then obtained. One of these historians asserts that persons flying from infected cities into the country carried with them the fomites of the disease; which was there propagated to others, though they themselves sometimes escaped. So much for Evagrius. Procopius avers that the attendants on the sick, and even those who buried the dead, often escaped, and that those of the attendants who took the disease, did so from fatigue and drudgery.

Cedrenus, while giving an account of a pestilence that issued (as was the opinion) from Ethiopia, and ravaged Europe and the East for fifteen years, is remarkably particular respecting *fomites*, as a mode of propagating the disease. He affirms that the subtle poison, or contagious matter, lurked in the garments of those who had been affected with the pestilence, and thence induced it in others. And yet after such testimony as this, Dr. Maclean and his followers will triumphantly tell you that no idea of contagion was entertained till the translation of the Council of Trent. They will tell you this over and over again, knowing it to be—*the reverse of truth.*

Dr. Marx quotes several passages from Aretæus in proof of his belief in contagion. Some of them are equivocal—one at least is convincing. Speaking of elephantiasis, he characterises the disease as not only frightful and loathsome to behold—but also *as contagious as the plague.* Galen is also quoted by our author; but not much to the purpose. This venerable father in the healing art evidently considered several diseases contagious, and among others consumption; but his opinions are mixed up with such absurdities as to produce but little effect on the unprejudiced mind.

Paul of Ægina follows Aretæus, in considering elephantiasis *as infectious as the plague.* From this time the doctrine of contagion appears to be lost sight of for many centuries among philosophers, historians, and physicians. The reasons which have been assigned for this mysterious silence are quite unsatisfactory—indeed, we consider the hiatus as incapable of explanation at this period.

In turning to the Roman authors, Lucretius is the first examined by Dr. Marx. His doctrine approaches very nearly to the most general conviction of the present day—namely, that pestilential diseases arise, at first, from changes in the air or exhalations from the earth, and afterwards, when fully established or epidemic, that they are propagated by contagion. So Ovid.

"Corpora fœda jacent, vitiantur odoribus auræ

Adflatuque nocent, et agunt contagia late.

Quo propior quisque est, servitque fidelius ægro,

In partem leti citius venit." *Metamorp.*

It must be confessed, however, that the language of poets is not that which we would wish to use in arguments of this kind. The historian is a more respectable authority than the poet.

Livy's testimony is the most unequivocal of all. Speaking of the epidemic at the siege of Syracuse, he says (what indeed we quoted many years ago ourselves) "et primo temporis ac loci vitio et ægri erant, et moriebantur: postea *curatio ipsa et contactus ægrorum vulgabat morbos.*" Nothing can be more complete testimony than this.

We must pass over a host of Latin writers, in order to advert to the testimony of Cœlius Aurelianus, an accomplished and able *physician*, who treats at great length on contagion and contagious diseases. This author considers elephantiasis, hydrophobia, and pestilence, as contagious. Some other testimonies are adduced from the Arabian physicians, requiring no notice in this place.

In respect to the state of the question in the middle ages, and for some time after the revival of learning, Omodei is the author to be consulted by the curious.

Muratori informs us that, in 1340, a pestilence prevailed in Italy, and was supposed to be imported from Egypt, Syria, or Greece. There were no quarantine laws in use then—at least no effectual means of preventing the introduction of contagious matters. In 1348, another epidemic ravaged Florence, Bologna, and some other places, its origin being attributed to importation from the Levant, in Venetian and Genoese vessels. From these countries, it is said to have passed into France, Germany, and England. It was at this time that precautionary measures began to be put in force. Numerous authorities are here produced to shew that the plague of 1348 was propagated by contagion, and by articles worn or handled by the sick—that is by *fomites*. Against this plague, the governor of Venice put in force several police regulations—which were afterwards imitated by many other states and cities.

It was in the year 1481, that Marselius Ficinus wrote his *Consilium Contra Pestem*, in which the modes of purification and prevention are fully treated of—and that upon the authority of ancient and modern physicians, of which his father was one. In those days, there were anti-contagionists, as well as in our own. Thus Varignana, a Professor of Medicine at Bologna, denied any contagious property, not only in plague, but in small-pox and measles! Gentilis, not so fortunate as Maclean, died of plague, denying its contagious character with his last breath! At the same time, it is proper to state that most medical writers of this period appear to have considered the cause of plague as a common evil to which all were exposed by living amidst corrupted air. There were exceptions, however, as in the case of Raymond da Vinario, who was an eye-witness of several epidemics in the city of Avignon, in the 14th century. In a manuscript on plague, left by this physician, he states it to be dangerous to have intercourse with the sick —or to approach those who come from infected places. He says that a single individual may infect a whole family, or even a city.

But some of the precise notions of contagion, as entertained in the present day, appear to have originated with Professor Forli of Padua, who died in 1413. He considered as contagious the pestilent diseases derived from touching the sick or their clothes—and recommends the magistrates to cause them to be removed, and put under the charge of old women not very apt to receive the infection.

We shall quote but one more authority for the belief in contagion prior to the date assigned for such belief by Dr. Maclean. This is Alexander Benedict, who taught medicine at Padua and Bologna. He published a Treatise on Pestilence in 1493, in which he maintains that the disease may be received by touching patients, and moreover affirms that the morbid principle is imbibed and retained in articles of dress, &c. used by the sick. Besides the separation of the sick, therefore, and the cutting off communication between them and the well, he recommends the purification of all articles of clothing which have been touched by the infected persons—an admonition not distinctly given by any physician previously.

We have now waded through a sea of researches, accumulated by the industry of two learned foreigners, presenting a very small percentage of their labours, which we thus place on record, rather as matters of curiosity than of any real utility. We never, indeed, could see the very great importance of referring back to the ancients for evidence of that which passes before our own eyes. There is no man of any practice in this country, who has not seen fever prove contagious—nor any of the present generation who have been in plague countries, who have not seen the same thing of plague, not even excepting Dr. Maclean himself, as the seams in his groins to this day testify.

It is almost needless to say, that the assertions of the last mentioned writer, respecting the origin of the doctrines of contagion, are now contradicted by a host of evidence quite irresistible.

---

2. *Coroner's Inquest—St. George's Hospital.* We have long been inclined to believe that Candide was right in his creed of Optimism. We do not agree with Pope, that "whatever is, is right"—but we firmly believe that there is no evil permitted in this world, without its attendant good—however invisible that good may sometimes be to short-sighted mortals. This view of the matter is consistent with the general benevolence of the Deity—and with the merciful dispensations of Providence, as manifested by the ever-varying events of the world around us. But although we may reverence the wisdom of our Creator in permitting evil agents to work good, it certainly is not to be expected that we should love or approve those agents themselves. The tornado which clears the air of pestilence, and the earthquake which gives vent to subterranean fires, are objects of horror in themselves, however beneficial in their consequences; and it is just so, though on a smaller scale, with those evil agitators and dark maligners who go about endeavouring to disturb the peace of society, and traduce the characters of their fellow-creatures, for the most selfish or wicked purposes, though with the ultimate effect of putting honest men on their guard, and thus preventing even the approach to error or neglect. Such maligners we must regard as evil spirits permitted to prowl on earth for the general benefit of mankind. They do good against their will. By their endeavours to calumniate merit, they render it more conspicuous; while the very attempt has a tendency to repress delinquency in other quarters.

We have been led into these reflections by a recent note of alarm sounded by some of these agitators, with a libellous accusation against one of our public institutions. A gentleman's servant, having met with an accident, was sent to St. George's Hospital, where he died five or six weeks afterwards. The minds of his master and fellow servants, who occasionally visited him, were poisoned by some idle reports or false impressions in the Hospital, and in consequence of this, a Coroner's Jury, in the plenitude of passion and ignorance, pronounced a verdict that reflected not only on the skill of the surgeons, but on the humanity and cleanliness of the nurses of the hospital. This, of course, produced a considerable sensation in the public mind, and the accusers thought they had effected their object of throwing odium on the hospital and its officers. But an open committee of the governors having sifted the whole transaction to the bottom, the frivolous and vexatious nature of the charge was so completely established, that the principal prosecutor (the master of the deceased, who had been led astray in the first instance) presented the Hospital with *one hundred pounds*, as an atonement for the outrage which he had, under false impressions, committed on the feelings of the surgeons who had the charge of the patient. Several others, we understand, who joined in the cry, have since made pecuniary expiation of the errors into which they were led.

Now, although it is evident that, in this instance, as in almost all others, good has sprung from evil, yet we think a lesson has been taught the medical officers of our public institutions, which it behoves them still further to profit by, for the sake of their own characters, and the good of society at large; we mean the propriety, not to say the necessity, of keeping an accurate and authentic registry and history of every important case which is treated within the walls of a public hospital. In every institution of this kind, there are intelligent pupils who are perfectly willing and able to keep these records—and we do maintain that such documents would at once supersede all idle gossip and malignant misrepresentations, which we have seen to be admitted as evidence on a coroner's inquest, and published in the daily prints, to the disgrace of all medico-legal proceedings in an enlightened age and nation.

But independent of the above considerations, the interests of medical science, and the good of the community, imperiously demand that the practice of our public hospitals should be rendered as extensively useful as possible, through the medium of *authentic reports*. This is no Utopian proposal. We see it in full and beneficial operation in other countries; and the rising spirit of inquiry will force its adoption in every country of Europe. Much as we detest the malignant and the false *reports* which have lately been spread respecting certain hospitals, we confess that we are not sorry to see the temporary evil, because we are convinced that it will accelerate the ultimate good. Are British physicians and surgeons afraid or ashamed of having their practice recorded and laid open to every eye? We hope and believe they are not. Then, why should they not follow the example of their Continental brethren, and benefit the profession and society at large by faithful hospital reports? We tell them again that, sooner or later, they will be *forced* to adopt this measure, and the sooner they do it, the sooner they will break the chain of misrepresentation by which they are daily dragged before the public, to the injury of their own characters, and the disgrace of the profession.

We cannot drop this subject without animadverting on the conduct of some individuals of the medical profession who seem ever ready to fan the flame of popular accusation against our public institutions and their officers. Such conduct is calculated to injure the profession generally in the eyes of the world—and assuredly is not the way to raise their own character. None, even of the most ignorant, will give them credit for any other motives than jealousy of others, or the blind hope of bringing themselves into notice. Blind hope, indeed! such notice will only illuminate their own disgrace.

P. S.—After the above was printed, we read the remarks of "a disinterested physician," on the aforesaid inquest, in the last No. of the Medical Repository. While we agree with this writer in many of his observations, we differ from him, toto cœlo, in one important point. He maintains that a Coroner's Jury has no business with the medical treatment of a patient in an hospital; we, on the other hand, contend that the jury has power to inquire into, and comment upon, every thing that has occurred to a patient, from the time he receives the accident or other cause of death, till the death itself. We shall put this in a clear point of view by an example.

A. receives a blow from B. in a scuffle, and is carried into an hospital. The surgeon prescribes a black draught, and C. the house-surgeon, or apothecary, from negligence or inattention, sends a phial of laudanum, which A. drinks, and dies in 24 hours. The Coroner's Jury is summoned, and they are told that they have nothing to do with the treatment of B. while in the hospital. They, therefore, find that the deceased died in 24 hours after a blow from B. and they bring in a verdict of homicide, or perhaps murder. Here we see what mischief would follow from the doctrine of "a disinterested physician." A charge of homicide would be wrongfully brought against B. while C. by whose negligence the man was poisoned, would be screened from all censure. Is it not manifest that the jury had the power of enquiring into the treatment of B. at the hospital, and of giving their verdict—"died by poison administered from negligence by C."

We are well aware, indeed, that, from the nature of a Coroner's inquest, and the imperfect evidence produced there, the verdict is very often wrong. We see this every day. Nothing is more common than to find the Coroner's Jury bring in a verdict of *wilful murder*, which is afterwards proved to be only homicide of the least culpable kind. But the people of an hospital have no exemption from such occurrences, and must participate the danger of them with the rest of the community.

---

3. *New College of Physicians.* This elegant new edifice opened with great splendour on the 25th of June last, there being present several Members of the Royal Family, many of the nobility, and, in short, a large concourse of the most distinguished personages, professional and non-professional. The Oration was delivered by the President, Sir Henry Halford, bart. with much dignity, and though conveyed in a dead language, which it is always difficult to follow in a speech, it evidently produced a considerable effect on the audience—especially that part of the Oration which alluded to the late, and ever to be regretted Dr. Matthew Baillie. We think we shall best illustrate the matter and manner of the President's Oration by extracting this passage, to which we take the liberty of annexing a liberal translation, for the benefit of those who are not accustomed to the style in which the Oration is constructed.

"In hoc dilecto nomine fas sit mihi commorari paulùm, et dolere, quòd huic excellenti viro, tot annos in eâdem nostrâ illâ laboriosissimâ vitæ ratione comiti, socio, amico, singulari in hanc domum pietate, hisce comitiis celebrioribus, huic solemnitati, huic illustrissimorum et nobilissimorum Hospitum cœtui non licuerit interesse: quanquam eum famæ satis diù vixisse scio, æternæ felicitati, quod humillimè spero, benè satis. Et enim, patre usus pio, à primâ usque adolescentiâ in explorando corpore humano fuerat versatissimus; et ex hâc studiorum ratione sapientiam et potentiam Dei maximâ admiratione, summâ veneratione contemplatus est. Posteà verò, cùm ad medicinam exercendam se accinxisset, facilè sensit, quantulùm corpori, morbis et ægrâ valetudine laboranti, subventurus esset Medicus, nisi qui animi quoque motus, vires, adfectus, perciperet: animi, scilicet, unius et ejusdem cum corpore, tamen diversi,—consociati cum illo, sed distincti,—in ejus compagibus inclusi et involuti, nihilominùs tamen liberi—immortale quid perpetuò præsentientis atque præmonentis, et illud futurum cupientis, tamen et metuentis. Ab his contemplationibus potentiæ ac majestatis divinæ ad debitum numini cultum præstandum incitatus est, ad fidem in Deo habendam, et ad totum se ei submittendum. Hinc pia illa vivendi regula, hinc spectata integritas. Hinc illi omnia graviter, humaniter, amabiliter mos erat cogitare;—hinc, quod cogitaverat, planissimè ac verissimè dicere;—hinc nihil alteri facere, quod sibi faciendum nollet;—hinc candor, caritas:—sed me reprimo; quanquam haud vereor, Optimates, ne vobis in præstantissimi hujus viri laudibus longior fuisse videar: quippe vestrûm quamplurimi sanitatem ejus judicio et consiliis acceptam refertis. Nec timeo, ne mihi succenseatis, Socii, quòd eum his saltèm accumulaverim donis, qui tantum sibi vestrûm omnium amorem vivus conciliaverit; qui industriæ, benevolentiæ, sanctitatis, innocentiæ exemplum (quod omnes utinam imitemur!) reliquerit." 15.

*Translation.* "And here I would pause, for a moment, on that beloved name (M. Baillie),—here express my grief that he, all excellent as he was—he, so many years of a laborious life, my companion, associate, friend—should have been doomed never to see the dedication of this place—never to mingle with this concourse of illustrious guests! He has gained fame on earth—and, I confidently hope, eternal felicity in Heaven. From his earliest years he had been engaged in the study of the human fabric, and, from this study he learnt to look with wonder and veneration on the power and omniscience of his God! But when he entered on the duties and practice of his profession, he knew right well how little—how very little, the physician could assist the efforts of the body to throw off disease, unless he were thoroughly versed also in the motions, the powers, and the affections of the mind—of that mind, I say, which is one and the same with the body, and yet different—associate of the body and yet separate and distinct—shut up and knitted in its frame work, yet free and unconfined—ever 'longing after immortality,' ever looking forward to that 'inevitable bourne,' yet glancing back with instinctive awe and fear! By this unceasing contemplation of the power and majesty of the Creator, he was taught to revere his commands—to place a firm reliance in him; and submit himself entirely to his parental care. Hence that pious life—hence that tried integrity—hence that constant habit of saying, in simplicity and truth, what he thought—of doing unto others as he would they should do unto him—hence his truly candid and charitable disposition. But I must restrain my feelings, although I never could dwell long enough on the virtues and merits of that honoured name—a name which will not be considered as overpraised by you, many of whom, perhaps, owe your health and existence, at this moment, to his consummate skill and judgment. I cannot fear that you will reproach me with adulation of BAILLIE, dead, who, when living, was in the hearts of all:—who has left behind him a most ennobling example (may we all imitate it) of industry, benevolence, piety, and innocency of heart." 15.

The whole oration is written in pure classical latin, though sometimes rather abstruse. We are very glad that the President took so favourable an opportunity of repelling the stigma of scepticism and irreligion, which the world at large is disposed to affix on the Medical Profession. This testimony as to the sentiments of a man who was deservedly at the head of his Profession—and coming as it now does, from such authority, will have a beneficial effect on the public at large, and also on medical men themselves. It was on this account that we selected the passage in question, in order that we might give it every possible publicity.

